# FAST (Flexible Acetylcholine Sensing Thread): Real-Time Detection of Acetylcholine with a Flexible Solid-Contact Potentiometric Sensor

**DOI:** 10.3390/bioengineering10060655

**Published:** 2023-05-27

**Authors:** Farbod Amirghasemi, Ali Soleimani, Shahd Bawarith, Asna Tabassum, Alayne Morrel, Maral P. S. Mousavi

**Affiliations:** Alfred E. Mann Department of Biomedical Engineering, Viterbi School of Engineering, University of Southern California, Los Angeles, CA 90089, USA; amirghas@usc.edu (F.A.); alisolei@usc.edu (A.S.); bawarith@usc.edu (S.B.); atabassu@usc.edu (A.T.); morrel@usc.edu (A.M.)

**Keywords:** acetylcholine, neural probe, electrochemical sensor, flexible sensor, neurotransmitter, cholinergic signaling, Alzheimer’s disease, solid-contact potentiometric sensor

## Abstract

Acetylcholine (ACh) is involved in memory and learning and has implications in neurodegenerative diseases; it is therefore important to study the dynamics of ACh in the brain. This work creates a flexible solid-contact potentiometric sensor for in vitro and in vivo recording of ACh in the brain and tissue homogenate. We fabricate this sensor using a 250 μm diameter cotton yarn coated with a flexible conductive ink and an ACh sensing membrane that contains a calix[4]arene ionophore. The exposed ion-to-electron transducer was sealed with a 2.5 μm thick Parylene C coating to maintain the flexibility of the sensor. The resulting diameter of the flexible ACh sensing thread (FAST) was 400 μm. The FAST showed a linear response range from 1.0 μM to 10.0 mM in deionized water, with a near-Nernstian slope of 56.11 mV/decade and a limit of detection of 2.6 μM. In artificial cerebrospinal fluid, the limit of detection increased to 20 μM due to the background signal of ionic content of the cerebrospinal fluid. The FAST showed a signal stability of 226 μV/h over 24 h. We show that FAST can measure ACh dynamics in sheep brain tissue and sheep brain homogenate after ACh spiking. FAST is the first flexible electrochemical sensor for monitoring ACh dynamics in the brain.

## 1. Introduction

**Figure 1:**
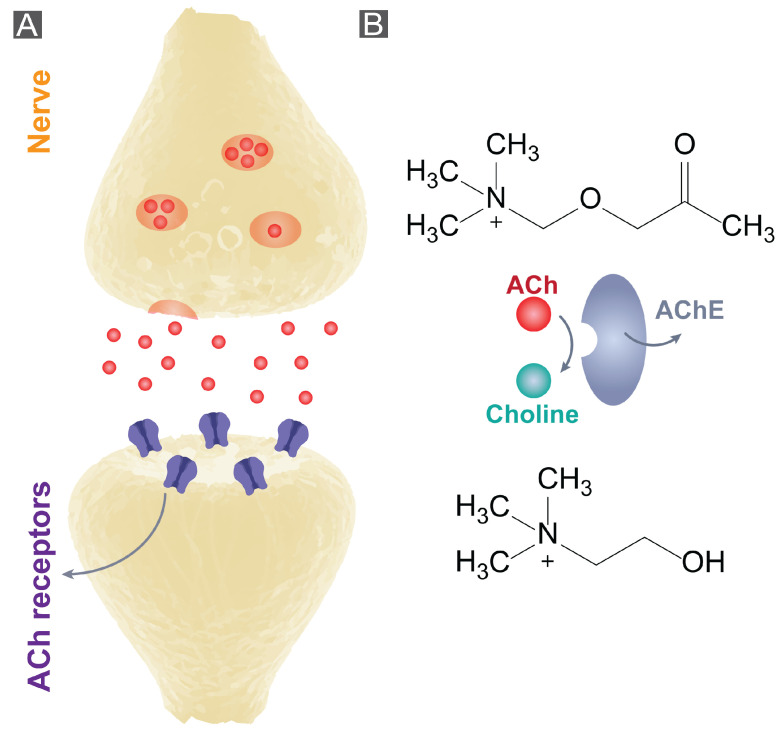
Acetylcholine detection. (**A**) Synaptic cleft of an active neuromuscular junction. (**B**) AChE degrades ACh to choline.

Acetylcholine (ACh) is a positively charged small molecule neurotransmitter that can be found in the central and peripheral nervous systems. ACh has essential roles in the body including learning, memory, motivation, and muscle control [1,2]. Disturbance in the physiological levels of ACh in the brain has been linked to dementia, Alzheimer’s disease, and psychiatric disorders, as well as anxiety and depression [3,4,5,6]. Tools for the selective sensing of ACh are crucial for a fundamental biological understanding of the role of this neurotransmitter in disease progression, as well as the rational design and testing of therapeutics that interact with cholinergic receptors. ACh has a wide physiological concentration range of nM to mM, with a short lifetime (half decay time of 2 ms [7]) due to rapid hydrolysis by acetylcholinesterase (AChE), see Figure 1. Therefore, effective ACh sensing tools should have fast response times, as well as a high selectivity and a good spatial resolution to gain physiologically relevant information on ACh dynamics. The most common method of ACh analysis is sampling of cerebrospinal fluid with a microdialysis probe, followed by analysis using mass spectrometry coupled with liquid or gas chromatography. Although a low limit of detection (LOD) can be achieved with this method, the temporal resolution (10 s) is insufficient for understanding concentration dynamics and requires sample collection and offline analysis [8,9,10,11,12]. This method is unsuitable for measurements on awake behaving animals due to the complexity of sampling. Moreover, the need for access to a mass spectrometer and method development diminishes the accessibility of this method for a broad range of researchers.

Several imaging tools based on magnetic resonance imaging [13] and fluorescence imaging [14] for ACh detection have been developed, including (1) genetic probes based on activation of G protein-coupled receptors [15], (2) a DNA-based enzymatic nanosensor [16], (3) selective quenching (on and off) based on the interaction of ACh and dyes and nanoparticles such as carbon quantum dots, calixarenes, and gold nanoparticles [14,17,18,19], and (4) use of nanoparticles modified with AChE for surface plasmon resonance (SPR) and SPR-enhanced energy transfer for imaging [20,21,22]. While each imaging technique has its pros and cons in terms of selectivity, sensitivity, response range, and response time, limited optical access to deep brain structures without implantation of bulky and destructive fiber optics remains a concern for this type of analysis. Electrical probes offer an alternative measurement as they can access deeper structures in the brain (while having inadequate spatial resolution compared to optical imaging methods) [23]. Improving the biocompatibility of neural probes has been the focus of research in recent decades [24,25,26,27,28,29]. Several groups have endeavored to fabricate flexible probes that can enhance the compatibility with the surrounding tissue and minimize tissue damage and subsequent scar tissue formation and rejection. Electrical neural probes have been fabricated on flexible substrates such as polyimide and parlyene, reducing the electrode size to tens of micrometers [24,25,26,27,28,29].

Designing electrochemical biosensors for ACh remains an active area of research [14]. Here, we have provided a summary of the recent work in Table 1. Unlike catecholamines, ACh is not electroactive and cannot be reduced or oxidized within the potential window of water. The majority of past work has focused on immobilization of AChE (to hydrolyze ACh to choline) and choline oxidase to convert ACh to hydrogen peroxide that can be detected using amperometric or voltammetric methods [14,30,31,32,33,34,35,36]. The main challenge with this type of biosensor is the limited lifetime of enzymes (especially for in vivo applications). In addition, the response time is limited by the kinetics of the enzymatic reaction and the time it takes to complete the voltage (or current) sweep [14]. Detection of local pH changes (using a potentiometric pH sensor on an electrode with immobilized AChE) has also been reported as an alternative readout method [37,38]. Different nanomaterials have been proposed to replace the enzymes and catalyze the electrochemical reaction of ACh at the electrode surface [39,40,41,42,43,44]. Limited specificity remains a concern for this type of sensor [14]. A distinctive view on electrochemical detection of ACh was proposed by us and others, where instead of relying on oxidation or reduction, the permanent positive charge of ACh is used to generate an electrical potential [14,45]. This category of sensors is known as potentiometric electrodes, where a sensing membrane and an organic receptor (ionophore) provide selectivity [46,47]. Potentiometric sensors have fast response times and read in the passive mode (do not consume ACh), which offers more advantages for real-time in vivo recording [45,46,47].

In prior work, we showed that Calix[4]arene can act as an ACh ionophore in a polymeric membrane liquid-contact potentiometric electrode [45]. We validated this sensor in mice brain homogenate [45]. Other works have followed by others where liquid-contact and solid-contact potentiometric sensors were fabricated using different materials and new ionophores [37,38,45,48,49,50,51,52,53,54,55,56,57]. He et al. reported a solid-contact ion-selective electrode (ISE) using a gold wire coated with a conductive polymer and an ACh selective membrane [58]. The developed ISE showed a sensitivity of 54.04 mV/dec and a detection limit of 5.69 μM. Chen et al. deposited PEDOT on a gold disk electrode and dropped the ACh selective membrane containing oxatub[4]arenes as an ionophore for ACh detection [48]. Ashmawy et al. deposited PEDOT/PSS on a carbon-screen-printed electrode and drop-casted the membrane on top of the PEDOT/PSS layer for ACh detection in serum [53]. All prior works in solid-contact ACh potentiometric sensors have used rigid substrates for electrode fabrication, which have large diameters that can cause severe tissue damage upon insertion in the brain.

In this work, we have developed the first flexible ACh potentiometric neural probe with a diameter of 400 μm. Probe flexibility and size are critical to minimize tissue scarring during implantation and readout. Matching the probe stiffness to that of brain tissue (dura matter for humans: 30–60 MPa and for rats: 0.1–2 MPa) is critical [26,59,60]. Early work on electrical probes focused on silicon or metal probes (Young’s modulus of 100–200 GPa) that caused substantial tissue damage. More recent works were performed on micropatterned flexible polymers such as polyimides (2–8 GPa) and parylene (3 GPa) [26,61]. To achieve a compact and flexible design for FAST, we utilized cotton yarn (similar stiffness to polymer neural probes [62]) as a substrate to hold the conductive ink and support the ACh sensing membrane. As a result of utilizing cotton yarn (250 μm diameter) to define the electrode width, there was no need for microfabrication steps in device fabrication. We showed the application of our flexible ACh sensing thread (FAST) for ACh analysis in artificial cerebrospinal fluid, sheep brain homogenate, and intact sheep brain tissue, and confirmed the real-time response of the sensor to changes in ACh concentration.

**Table 1:** A comparison of developed potentiometric sensors for acetylcholine determination. NR means not reported; NA means not applicable; MIP means molecularly imprinted polymer.

Electrode Specifics	Electrode Size	Flexible	Ion-to-Electron Transducer	Ionophore	Limit of Detection (μM)	Sensitivity (Slope, mV/dec)	Linear Range (M)	Biospecimen Used for Sensor Validation	Ref.
**Liquid-Contact Electrodes**									
Pulled-glass micropipettes	50.00 μm tip diameter	No	Ag/AgCl	*Acetylcholine dipicrylaminate*	20	59.4	10^−5^–10^−1^	striatum of rat	[49]
Membrane glued to a PVC tube	NR	No	Ag/AgCl	*Dioctyloctad*- *ecylamine*	5	52.92	10^−5^–8 × 10^−3^	NR	[52]
Commercial electrode body	NR	No	Ag/AgCl	*(allyloxy)*_12_ *cucurbituril[6]*	0.97	49.1	10^−6^–10^−3^	NA	[50]
Membrane glued to a PVC tube	0.5 cm tip diameter	No	Ag/AgCl	*Calix[4]arene*	0.008	52.92	10^−9^–10^−3^	rat brain homogenate	[45]
Commercial electrode body	NR	No	Ag/AgCl	*aryl-extended calix[4]pyrrol*	0.3	59.5	10^−6^–10^−2^	urine	[51]
Commercial electrode body	NR	No	Ag/AgCl	*Oxatub[4]arenes*	0.1	58.6	10^−6^–10^−2^	mouse brain homogenate	[48]
**Solid-Contact Electrodes**									
Commercial macrodisk electrode	NR	No	glassy carbon	Dibenzo-18-crown-6	10	NR	NR	NA	[54]
Commercial electrode body	NR	No	Carbon paste	β-Cyclodextrins	0.83	55.6	10^−6^–10^−2^	blood serum	[55]
Coated copper wire	NR	No	Graphite	*MIP/MAA*	4.5	55.2	10^−5^–10^−2^	NR	[56]
Coated gold wire	500 μm tip diameter	No	PEDOT:PSS	h-β-Cyclodextrin	5.69	54.04	10^−5^–10^−1^	synthetic serum	[58]
Solid graphite support	NR	No	polyaniline and carbon nanotubes	NA (MIP)	34.5	83.86	3.40 × 10^−5^–10^−3^	synthetic serum	[57]
Ceramic screen-printed	NR	No	PEDOT:PSS	*acetyl*-β-*cyclodextrin*	0.32	55.3	3.60 × 10^−6^–10^−3^	human serum	[53]
Carbon-coated cotton fiber	300 μm tip diameter	Yes	Carbon black	*Calix[4]arene*	2.6	56.11	10^−6^–10^−2^	sheep brain	This work

## 2. Materials and Methods

### 2.1. Chemicals and Reagents

Sodium tetrakis[3,5-bis(trifluoromethyl)phenyl]borate (NaTFPB), calix[4]arene (CX4), 2-nitrophenyl octyl ether (o-NPOE), high-molecular-weight poly(vinyl chloride) (PVC), tetrahydrofuran (THF, inhibitor-free, for HPLC), potassium hydrogen phosphate, potassium dihydrogen phosphate, D-(+)-Glucose monohydrate, potassium chloride, sodium chloride, calcium chloride, magnesium chloride, ammonium chloride, hydrochloric acid (HCl), γ-Aminobutyric acid (GABA), L-Glutamic acid monosodium salt hydrate, serotonin, dopamine, Acetylcholinesterase from Electrophorus electricus (AChE), and OmniPur Tris HCl buffer were purchased from Sigma Aldrich. We prepared all stock solutions with MiliQ deionized water (resistivity of 18.2 MΩ·cm) unless stated otherwise. The sheep brain tissue was purchased from a local market (Tehran Market, Los Angeles, CA, USA).

### 2.2. Electrode Fabrication

**Figure 2:**
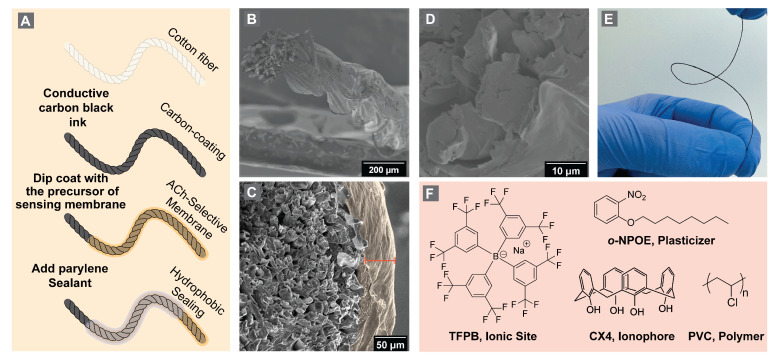
(**A**) Fabrication scheme, (**B**) SEM image of carbon-coated cotton fiber (scale: 200 μm) (**C**) SEM image of ACh-membrane-coated fiber electrode (highlighted area, scale: 50 μm), (**D**) SEM image of hydrophobic sealing (scale: 10 μm), (**E**) image of twisted conductive thread electrode, (**F**) molecular structures of ACh-selective membrane components.

Figure 2 illustrates the fabrication steps for the FAST sensors. First, we prepared a conductive ink by dispersing carbon black powder in a polymeric matrix. For this purpose, we fabricated an ink containing 25 wt.% carbon black and 75 wt.% organic polymer. The organic polymer matrix encompasses a polymer (PVC) and its plasticizer (o-NPOE). We dissolved approximately 1 g of the total ink mixture (1:2 ratio of PVC to o-NPOE) in 3 mL THF and stirred for 3 h. We cut a 6 cm long 100 % mercerized cotton fiber and dipped it in the conductive ink with an approximate resistivity of 300 Ω per one centimeter of the fiber, allowing it to dry for 3 h. Next, we formed the ACh-selective membrane precursor by adding 10 mg of NaTFPB (ionic site), 11 mg of CX4 (neutral ionophore), and 330 mg of PVC dispersed in 2.5 mL of THF containing 660 mg of o-NPOE. An ionic site ensures permselectivity for the electrodes and maintains a constant concentration of ACh^+^ in the sensing membrane. The role of each component in the sensing membrane and the theory of potentiometric sensors have been described in detail by Chen et al. [46]. The ACh-selective membrane precursor was stirred overnight until we distinguished a translucent membrane from starting-powder-containing precursor. Next, we added 5.0 μL of 10.0 mM acetylcholine chloride (to facilitate electrode conditioning and exchange of the ion exchanger salt with ACh^+^) and stirred for 24 h, where an opaque precursor formed.

We dip-coated conductive threads in the ACh-chloride-spiked ACh-selective membrane precursor for 30 s and repeated this step three times (covering an approximately 2.0 cm long sensing area). The electrodes were allowed to dry overnight to ensure evaporation of the organic solvent. We utilized a specialized parylene deposition system to deposit 5.0 μm thick parylene C for five hours (PDS 2010, Specialty Coating Systems Inc., Indianapolis, IN, USA) to encapsulate the middle section of the electrodes, leaving 1.0 cm of membrane and 1.0 cm of the conductive thread exposed on either end.

We utilized threads as a flexible and durable substrate for the sensing platform, where the threads provided a mechanical support for the conductive ink and the ACh^+^ sensing membrane. The fabrication of FAST sensors is straightforward and does not require a complex cleanroom setup. The thread itself defines the probe size. Threads are commercially available in various diameters, providing several options for making FAST sensors of the desired probe size. Utilizing carbon black as a primary component of conductive ink enabled us to increase the surface area of the sensors due to the porous nature of this material for the development of solid-contact potentiometric sensors. In prior work, we demonstrated that we can develop stable and reproducible yarn-based potentiometric sensors for organic and inorganic ions using our carbon black ink [63,64,65]. The SEM imaging illustrated that we could fabricate 250 μm thick sensors (Figure 2B). Moreover, the ACh-selective membrane adds approximately 50 μm to the thickness (Figure 2C). The parylene C coating also adds around 5.0 μm to the thickness of the fabricated sensors. Parylene C can provide pinhole-free water-proof encapsulation, without compromising the electrode stiffness. Parylene has an elastic modulus of 3 GPa, which is lower than the cotton substrate [26,61].

### 2.3. Measurement Protocols

We conducted potentiometric measurements by utilizing a 16-channel potentiometer (Lawson Labs, Malvern, PA, USA) controlled with EMF Suite 2.0 software (Lawson Labs). We used a commercial reference electrode (DX200, Mettler-Toledo, LLC., Columbus, OH, USA, Oakland, CA, USA) containing two inner filling solutions: 3.0 M KCl saturated with AgCl as the reference solution (inner filling solution) and 1.0 M lithium acetate as a bridge electrolyte. A Thermo Scientific Orion Star A211 pH kit was employed for pH adjustments. We built the calibration plots for ACh^+^ by a series of half dilutions. We implemented the IUPAC recommendations to determine the limit of detection (LOD), where we applied two linear regressions (electromotive force (emf) vs. logarithm of the primary ion concentration) within two concentration ranges. The first regression belongs to the region where the FAST sensors demonstrated Nernstian behavior to serial dilutions, and the second regression includes the concentration range where the emf of the ACh^+^ plateaued. The intersection of formed linear regressions determines the LOD for the FAST sensors [45,66,67].

## 3. Results and Discussions

FAST is an all-solid-state potentiometric sensor for direct detection of ACh^+^ in the brain. FASTs detection mechanism relies on a forming phase boundary potential at the interface of the ACh-selective membrane and positively charged ACh ions in the aqueous phase based on host–guest chemistry [46]. We utilized a flexible thread as an ion-to-electrode transducer to fabricate an all-solid-state ACh^+^ sensing thread, where an ACh-sensing polymeric membrane coated the conductive thread substrate to create a flexible and fine solid-state ACh sensor (Figure 2). We validated the sensitivity and selectivity of the FAST platform in artificial cerebrospinal fluid (aCSF), sheep brain homogenate, and sheep brain tissue.

### 3.1. FAST Response: Sensitivity and Selectivity

The all-solid-state potentiometric FAST sensor operates based on forming a nano-scale charge separating layer between the aqueous sample and the organic membrane [46]. We created our experimental setup by utilizing a double-junction external reference electrode and the FAST sensor, where we positioned the electrodes in the aqueous sample solution containing ACh^+^. Here, we particularly focused on developing a flexible ACh^+^ sensor itself. Therefore, all measurements were performed by employing a commercial reference electrode. We measured the FAST sensor’s corresponding electromotive force (emf) values under zero-current conditions. The Nernst equation, Equation (1), governs the correlation between emf potential and ACh ion activity in the sample solution [46].

(1) $$emf=E{}^{\circ}+\frac{RT}{z_{i}F}ln\left(a_{ACh}+\sum k_{ACh,j}^{pot}a_{j}^{z_{A}Ch/z_{j}}\right)$$

where *a*${}_{ACh}$ is the activity of the *ACh*${}^{+}$ ion and R, F, and T represent the universal gas constant, Faraday constants, and apparatus temperature, respectively. $k_{i,j}^{pot}$ is the selectivity coefficient that quantifies the selectivity of the sensor for the primary ion *i* over the interfering ion *j*.

**Figure 3:**
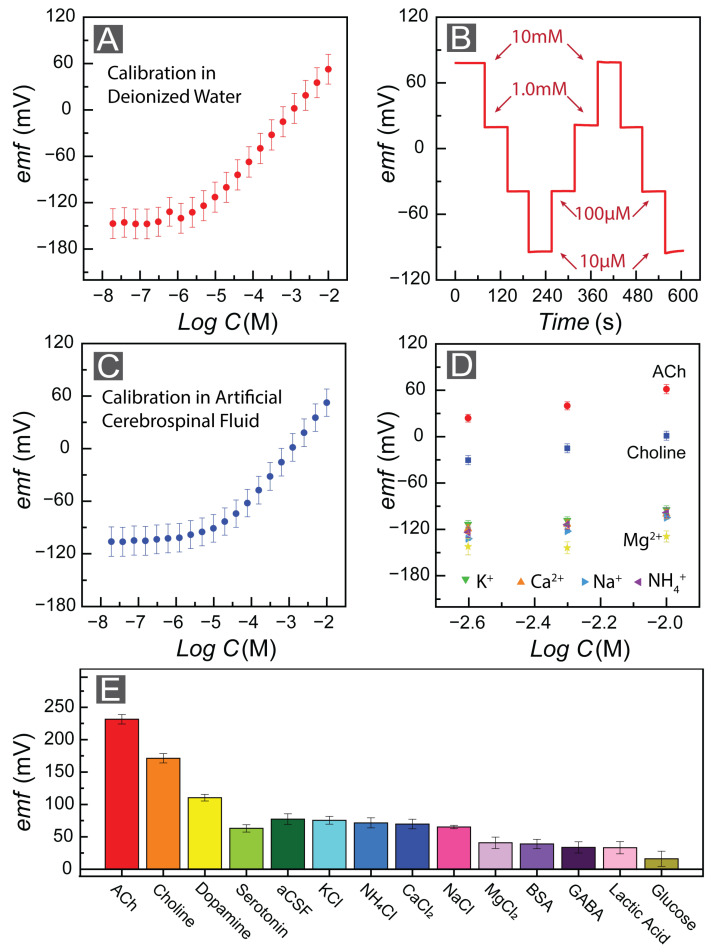
(**A**) Potentiometric response of pristine FAST sensors in DI water containing ACh with concentrations ranging from 10^−8^ M to 10^−2^ M. (**B**) Stable emf response of FAST with various ACh concentrations. (**C**) Potentiometric response of FAST in aCSF containing ACh with concentrations ranging from 10^−8^ M to 10^−2^ M. (**D**) Biased response of FAST in the presence of interfering ions (Acetylcholine (•), Choline (■), Potassium (▼), Sodium (▶), Ammonium (◀), Calcium (▲), Magnesium (♦)). (**E**) Selectivity of FAST against various electrolytes (10.0 mM) and physiologically relevant biomolecules (BSA: 1.6 mg/mL, serotonin: 3.0 μg/mL).

We measured the potentiometric response of ACh^+^ which was generated at the interface of the ACh-sensing membrane and the aqueous ACh^+^ solution (Figure 3A). Our sensing membrane is composed of a polymer matrix to provide mechanical support. The ACh^+^ selective membrane contains approximately 1.0 wt.% hydrophobic ions with a negative charge and 0.8 wt.% CX4 (ionophore), which enhances selective binding to the ACh^+^ ions [45]. The emf values at the interface of the membrane/sample solution directly correlate with the activity of the ACh^+^ ions in the membrane and the sample solution [46,47].

We tested the performance of FAST by quantifying the sensitivity of the FAST sensor toward ACh^+^. To do this, we constructed a calibration curve, where we plotted the emf response of FAST in the ACh^+^ concentration range of 10.0 nM–10.0 mM with a DI water background. The FAST sensor (n = 3) illustrated Nernstian behavior, where the slope of the linear range was 56.11 ± 0.15 mV/d (Figure 3A), commensurate with the theoretical Nerstian value of 59.2 mV/d. Moreover, the LOD of the FAST sensor is 2.6 μM. We observed the emf trace over several orders of magnitude for the FAST sensors and they showed excellent signal stability (Figure 3B). Next, we conducted a calibration study in aCSF to be more physiologically relevant by mimicking cerebrospinal fluid as our target biofluid. Here, the FAST sensor (number of electrodes n = 3) illustrated a slope of 57.02 ± 0.32 mV/d with an LOD equal to 20.9 μM (Figure 3C).

Selectivity towards the primary ion of interest in the presence of interfering ions is an essential element of any sensing tool. Therefore, we quantified the response of the FAST sensors toward ACh^+^ ions against the most abundant interfering ions in various biofluids, including CSF. Here, we used the separate solution method (SSM) [66], as it is a frequently employed and well-established approach to determine the selectivity of potentiometric sensors. In this approach, we quantify the emf response of the FAST sensors within two distinct solutions, one comprising the ACh^+^ (primary ion only without the presence of an interfering ion) and the second solution containing only the interfering ion (*j*) at the same concentration (in the absence of primary ACh^+^ ion). If the repective emf responses for the primary ACh^+^ and interfering ion *(j${}^{Z_{j}}$)* are E${}_{ACh}$ and E${}_{j}$, we can utilize Equation (2) to calculate the selectivity coefficient [67,68]. We report the selectivity values of the FAST sensors based on the discussed SSM approach in Table 2 for electrolytes that are abundant within biofluids, including sodium, potassium, and calcium. Th calculated selectivity coefficients are comparable to reported values in other studies [45,53].

(2) $$K_{i,j}^{POT}=a_{i}^{\left(1-\frac{z_{i}}{z_{j}}\right)}e^{\frac{\left(E_{j}-E_{i}\right)\left(z_{i}F\right)}{RT}}$$

Furthermore, we assessed the FAST sensor’s selectivity towards physiologically relevant electrolytes and biomolecules by observing the emf response of the FAST sensors. We observed a much more robust response to ACh^+^ in comparison to the interfering electrolytes (10.0 mM) and biomolecules, including GABA (10.0 mM), lactic acid (10.0 mM), bovine serum albumin (1.6 mg/mL), and glucose (10.0 mM) (Figure 3E). Dopamine and serotonin are among several neurotransmitters which could play a critical role in cholinergic activities of the brain [69,70]. However, dopamine and serotonin are electroactive, but they are not charged. Therefore, creating selective electrochemical sensors for these neurotransmitters has been studied for decades [71]. We also assessed the FAST sensors’ selectivity toward these neurotransmitters (dopamine 10.0 mM and serotonin 3.0 μg/mL). The corresponding emf response indicated that the interference of these biological molecules would not affect the FAST sensors’ performance (Figure 3E).

### 3.2. FAST Stability: Drift Analysis for Long-Term Measurements

**Figure 4:**
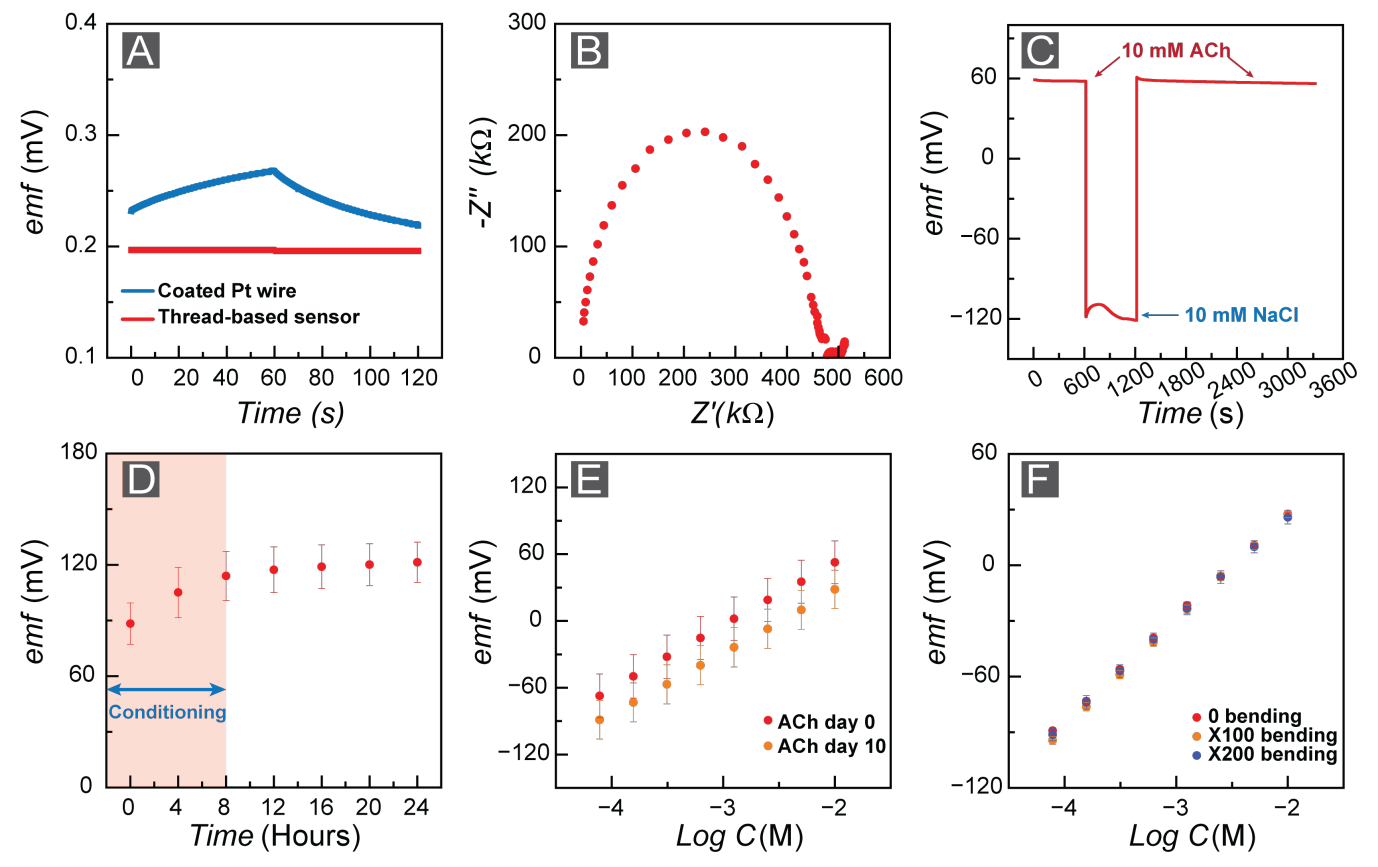
(**A**) Chronopotentiometric response of coated Pt wire (blue) and FAST sensor (red) to ±1.0 nA perturbations. (**B**) Nyquist plot to determine ACh-selective membrane resistivity. (**C**) Water layer test. (**D**) Long-term drift measurements (8 h of initial electrode conditioning). (**E**) Potentiometric response of FAST sensors after 10 days of storage (n = 3). (**F**) Potentiometric response of FAST sensors after 100 and 200 times bending (n = 3).

We aim to create an all-solid-state sensor to serve as a neural probe for in vivo studies. Therefore, the stability of the FAST sensor is a critical part of this study. For this purpose, we assessed the stability of our sensor in various scenarios. The stability of sensors is defined as the potential change over time (signal drift). First, we considered the correlation between the potential drift and the capacitance at the interface of the ion-selective membrane and the ion-to-electron transducer based on Equation (3), and we performed a chronopotentiometry experiment [72], where we applied a perturbation current of ±1 nA to the system and recorded the potential change over time. The chronopotentiometry test revealed 1 μV discharge of the capacitance over 60 s, which correlates to a 60 μF capacitance for the ion-to-electron transducer layer. To provide a comparison, Figure 4A shows a coated wire (Pt) ACh-selective electrode, where the ion-to-electron transducer has a small capacitance (1 μF) and thus a poor signal stability.

We also measured the resistivity of the ACh-selective membrane by performing electrochemical impedance spectroscopy (EIS), which yielded a 0.45 MΩ membrane resistivity (Figure 4B) by fitting data to an equivalent Randles circuit [73,74]. Next, we studied the water layer formation within the fabricated FAST sensors as a factor in all-solid-state ISE stability [75]. We monitored the emf response of the FAST sensors (n = 3) submerged in 10.0 mM AChCl for 10 minutes and then changed the solution to 10.0 mM NaCl while monitoring the emf for another 10 min. Lastly, we replaced the 10.0 mM NaCl solution with a 10.0 mM AChCl solution and continued to observe the emf values for 30 min. Based on the water layer test, we observed an approximately 0.4 mV/min drift in 10.0 mM NaCl solution, which indicates the possibility of water layer formation (Figure 4C). However, to assess the effect of ion exchange, we conducted prolonged drift measurements for pristine FAST sensors in 10.0 mM AChCl solution and monitored the emf response over a 24 h period. We allowed an 8 h period for electrode conditioning and then quantified the drift. We calculated a 226 μV/h (3.7 μV/min) drift for the conditioned FAST sensors (Figure 4D).

(3) $$i=C\frac{dV}{dt}$$

We assessed the performance of the FAST sensors to confirm their sensitivity after storage (Figure 4E). We repeated the calibration measurements for the same FAST sensors after storing them for 10 days in a ziplock bag. The sensitivity of the FAST sensors (n = 3) did not change significantly. We observed a sensitivity of 56.75 ± 0.18 mV/dec for the pristine electrodes, and re-calibrating the same sensors after 10 days indicated a sensitivity equal to 58.88 ± 0.81 mV/dec.

Fabricating flexible all-solid-state ISEs is one of the significant aspects of this study; to our knowledge, this is the first fabricated flexible all-solid-state ISE for ACh detection. Therefore, we performed a bending test on the FAST sensors to determine the impact of bending and other environmental conditions on the performance of the sensors. For this purpose, first, we calibrated pristine FAST sensors where we obtained a sensitivity of 55.63 ± 0.51 mV/dec. Then, we bent each electrode 180 degrees 100 and 200 times and calibrated them after each set, where we obtained sensitivities of 57.87 ± 0.63 mV/dec and 55.67 ± 0.41 mV/dec, respectively (Figure 4F). This experiment indicated that the developed FAST sensors are suitable for in vivo measurements.

### 3.3. FAST Feasibility: Application of FAST Sensors in Cerebrospinal Fluid

**Figure 5:**
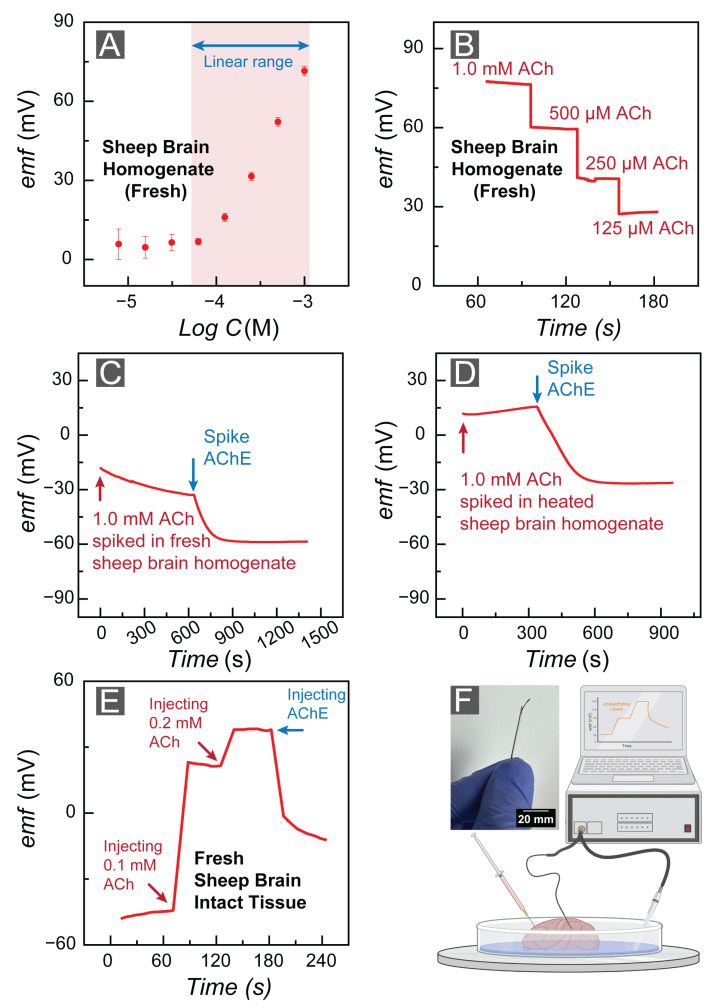
(**A**) Calibration curve of the FAST sensors in fresh sheep brain homogenate. (**B**) emf trace corresponding to the fresh sheep brain homogenate calibration. (**C**) FAST sensor’s response to the fresh sheep brain homogenate over time. (**D**) FAST sensor’s response to the heated sheep brain homogenate over time. (**E**) emf response of the FAST sensors to injection of ACh and AChE. (**F**) Experimental setup for testing the FAST sensor in brain tissue slices.

We examined the feasibility of the FAST sensors in biological samples. For this purpose, we utilized sheep brains due to them (i) recapitulating several aspects of the human brain physiology, (ii) being more acceptable in ethical committees, (iii) being readily available, and (iv) being less expensive. These factors make sheep advantageous as an experimental model for translational research among various animals [76]. We purchased the sheep brain tissue from a local butcher’s shop. We calibrated FAST sensors in fresh sheep brain homogenate. First, we measured the emf value in fresh brain homogenate to determine our ACh^+^ baseline. Next, we calibrated FAST sensors in the brain homogenate by spiking the homogenate to reach 1.0 mM ACh^+^ concentration (we performed a baseline correction). The FAST sensors illustrated a linear range with Nernstian behavior at concentrations ranging from 10${}^{-3}$ to 10${}^{-4}$, and the FAST sensors revealed a sensitivity of 57.32 ± 0.36 mV/dec (Figure 5A). Figure 5B depicts the FAST sensor’s response stability in fresh sheep brain homogenate.

We validated the sensitivity response of the FAST sensor by monitoring the emf changes in fresh brain homogenate. The existing acetylcholinesterase (AChE) in the fresh brain can hydrolyze ACh over time to form choline. Figure 5C illustrates the decreasing emf response of the FAST sensor in the fresh brain with a rate faster than our quantified drift (94,700 μV/h). Moreover, we prepared AChE by diluting 1.0 mg of AChE in 1.0 mL of fresh 20.0 mM Tris HCl (pH = 7.5) buffer. The addition of AChE to the homogenate indicated a faster degradation of ACh. We also examined the robustness of our experiment by heating the sheep brain homogenate for 4 h at 80 °C (to denature the AChE in the brain tissue) and monitoring the emf response of the FAST sensor. The emf corresponding to heated brain homogenate illustrated a steady emf response, validating that the previous decline in ACh response was associated with cholinergic activities of the sheep brain (Figure 5D). This experiment confirmed that the sensor emf changes were due to AChE in the brain tissue and ACh hydrolysis, and not caused by sensor malfunction.

We affirmed the feasibility of the FAST sensors by positioning them in fresh sheep brain slices floating in aCSF to mimic physiologically relevant conditions for in vivo recording. As the FAST sensors were too flexible to be directly inserted in the brain tissue, a 20 G × 1-1/2" hypodermic needle (Air-Tite Products Co., Inc., Virginia Beach, VA, USA) was used as a guide for sensor insertion in the intact brain tissue (Figure 5F). Next, we monitored the baseline emf response of the FAST sensors for 60 s before spiking the brain slice with a 0.1 mM ACh solution. Injection of 100 μL ACh^+^ (0.1 mM) led to an approximate 60 mV emf increase, confirming the feasibility of the FAST sensors for in vivo studies. Moreover, we spiked the same brain slice with a 0.2 mM ACh solution and AChE to affirm the FAST sensor’s response.

## 4. Conclusions

This work demonstrates the fabrication of the first flexible electrochemical sensor for real-time evaluation of ACh^+^ levels in the brain. Sensor flexibility was achieved using ink-coated cotton fibers sealed with parylene C to achieve a probe that is less than 400 μM. Here, we validated the performance of the FAST potentiometric sensor for detecting ACh^+^ levels ranging from 62.5 μM to 1.0 mM in brain homogenate. The response time was on the order of a few seconds by utilizing hydrophobic doping of ACh-selective membranes with calix[4]arene to enhance the selectivity and the biased response of the FAST sensors towards ACh^+^.

The FAST sensors successfully detected ACh^+^ levels in fresh and heated sheep brain homogenates without any biosample processing or extraction steps. Moreover, the FAST sensors were capable of quantifying ACh levels in fresh sheep brain tissue. We developed the FAST sensing platform to enable more sustainable probing tools for ACh^+^ levels and to enable examination of cholinergic signaling pathways in in vitro and in vivo studies. However, the FAST sensing platform could serve as a precision tool for identifying new drug candidates for neurodegenerative disease management and assessing the enzymatic activities associated with cholinesterase enzymes in intricate biological matrices.

Future work is still needed to improve the FAST sensor for in vivo studies and long-term sensor implantation. Specifically, increasing the capacitance of the ion-to-electron transducer layer and increasing the long-term stability is a high priority. Moreover, the sensor showed water layer formation, which could be addressed by increasing the hydrophobicity of the ion-to-electron transducer. The sensor’s selectivity should be improved to achieve physiologically relevant measurements in complex brain tissue. This can be achieved by the design of better ionophores or by using innovative sensing matrices such as the fluorous phase [77,78,79]. 

## Figures and Tables

**Table 2:** The selectivity coefficients of FAST over potentially interfering ions (*j*).

Interfering Ions (*j*)	${\mathrm{logK}}_{i,j}^{\mathrm{POT}}\left(\mathrm{SSM}\right)$
Sodium	−2.81
Potassium	−2.63
Ammonium	−2.69
Choline	−1.02
Calcium	−3.73
Magnesium	−4.22

## Data Availability

Data are contained within the article.

## Abbreviations

The following abbreviations are used in this manuscript:

ACh	Acetylcholine
BSA	Bovine serum albumin
GABA	γ-Aminobutyric Acid
NA	Not applicable
NR	Not reported
emf	Electromotive force
ISE	Ion-selective electrode
MWCNT	Multi-wall carbon nanotube
f-SWCNT	Functionalized single-wall carbon nanotube
PEDOT:PSS	Poly(3,4-ethylenedioxythiophene) polystyrene sulfonate
ANI	Aniline
MIP	Molecular imprinted polymer
MAA	Methacrylic acid
KTpClPB	Potassium tetrakis(4-chlorophenyl)borate
bTPCX4p	Bis/Tetraphosphonatecalix[4]pyrroles
h-β-cyclodextrin	Heptakis(2,3,6-tri-O-methyl)-β-cyclodextrin
CX6-HEA	Calix[6]arene-hexaethylacetate
